# Effects of a high-power laser eye exposure on avian foraging behaviour: implications for the safety of laser bird deterrents

**DOI:** 10.1093/conphys/coag004

**Published:** 2026-02-05

**Authors:** Arden Blumenthal, Deona Harris, Bret A Moore, Edward F Melvin, Esteban Fernández-Juricic

**Affiliations:** Department of Biological Sciences, Purdue University, 915 W. State Street. West Lafayette, IN 47907, USA; Department of Biological Sciences, Purdue University, 915 W. State Street. West Lafayette, IN 47907, USA; Department of Small Animal Clinical Sciences, University of Florida, College of Veterinary Medicine, 2015 SW 16th Ave., Gainesville, FL 32608, USA; School of Aquatic and Fishery Sciences, University of Washington, 1122 NE Boat St, Box 355020 Seattle, WA 98195-5020, USA; Department of Biological Sciences, Purdue University, 915 W. State Street. West Lafayette, IN 47907, USA

**Keywords:** Binocular vision, birds, eye injury, foveal vision, laser deterrents, pest management, visual foraging

## Abstract

To prevent human–bird conflict, lasers have been developed as nonlethal wildlife control methods despite being known to cause eye injury in humans. However, little is known about how much laser exposure can affect visually driven activities critical for survival, such as foraging. We assessed how laser exposure and its output energy affected avian visual exploratory behaviour during foraging and food consumption. We exposed house sparrows to a high-energy laser (Seabird Saver) under controlled conditions and measured within Week 1 after exposure and within Week 2 after exposure their foraging behaviour when visually locating millet seeds against a high contrast (easy task) and low contrast (difficult task) background. We found that house sparrows arrived at the food patch quicker and decreased their use of binocular vision within Week 1 after exposure compared to before exposure. Within Week 1 and within Week 2 after exposure, birds changed their rates of scanning depending on the difficulty of the foraging task. They also developed laterality by increasing foveal (i.e. high acuity) visual exploration rate using more the left compared to the right eye, particularly with increasing laser energy levels. Laser exposure increased pecking rates and seed consumption rates both within Week 1 and within Week 2 after exposure. This result is consistent with variations in body mass, which decreased markedly right after laser exposure, but animals recovered their pre-exposure weight over a few days. Our findings suggest that exposure to a high-energy laser (Seabird Saver) can alter visual exploratory behaviour in the context of foraging and influence foraging effort and food consumption rates. We discuss the implications for the use of lasers as wild bird deterrents at airports, landfills, fisheries, etc.

## Introduction

Negative interactions between humans and birds can cause property damage and destroy crops, leading to monetary loss (De Grazio, 1978; Allan, 2000). Different types of bird deterrents (lethal and non-lethal) have been developed to manage populations of different species. Lethal control methods have been declining in popularity as they are controversial with the public and even non-effective for long-term management (Dolbeer, 1998; Cook et al., 2008; Linz et al., 2015). Non-lethal deterrents, which employ an aversive stimulus in often a single sensory modality, are preferred (Rivadeneira et al., 2018; Enos et al., 2021).

A non-lethal wildlife deterrent that is growing in popularity is the laser (light amplification by stimulated emission of radiation). Lasers have some advantages: they do not make noise, can target a specific area over a long distance, can be made in various colours and can be used around various man-made structures (Blackwell et al., 2002; Gilsdorf et al., 2002; Cassidy, 2015). At airports, lasers are used to repel nesting, feeding or flocking birds (Blackwell et al., 2002; Briot, 2005; Baxter, 2007). Lasers have also been used to successfully repel other bird species from windmills, shipping vessels, oil sands and crops (Marques et al., 2014; Cassidy, 2015; Dredge, 2017; Brown and Brown, 2021; Manz et al., 2024; Sieving et al., 2025).

Laser safety standards, developed from research on mostly mammals, have been used to determine laser injury thresholds and establish safe viewing conditions for human eyes and predict damage from a given laser exposure (Zwick, 1984; Schmeisser, 1996). The degree of damage depends on the energy delivered to the eye (Campbell et al., 1966; Peppers and Hammond, 1969; Hudson, 1998; Barkana and Belkin, 2000), which is determined by the power output of the laser (Powell et al., 1971; Ham et al., 1979a), the size of the laser beam on the retina (Robbins and Zwick, 1999; ICNIRP, 2000; American National Standards Institute, 2014; Lund et al., 2014; Wang et al., 2014), the length of exposure to the laser (Peppers and Hammond, 1969; Ham et al., 1979b) and the wavelength of laser light (Ham et al., 1976; Ham et al., 1979a, 1979b; Chen et al., 2011). The energy density of the laser exposure on an eye increases with increasing laser power, longer exposure time and smaller beam diameter per a given power (ICNIRP, 2000; Ziegelberger, 2013a, 2013b; American National Standards Institute, 2014).

Based on mammalian studies, laser-eye exposure has the potential to result in ocular injury; more specifically retinal lesions characterized by cell death, disruption of cellular layers, hypopigmentation, haemorrhage or even retinal detachment (Birngruber et al., 1983; Leibu et al., 1999; Belokopytov et al., 2010; Lee et al., 2014; Xu et al., 2016). Laser-eye exposure can also cause functional loss of vision, such as temporary or permanent loss of visual acuity (Glickman et al., 1996; Zwick et al., 1997, 1999; Robbins and Zwick, 1999; Lee et al., 2014), decrease in the ability to distinguish objects of similar luminance or decreased contrast sensitivity (i.e. ability to distinguish an object from the background based on luminance) (Gunduz and Arden, 1989; Glickman et al., 1996; Zwick et al., 1999), decreased colour discrimination (Robbins et al., 1980; Robbins and Zwick, 1996) and impaired ability to track objects (Stuck et al., 1996).

How laser light specifically affects avian eyes is for the most part unknown (Glahn et al., 2000). We should expect avian eyes to be potentially vulnerable to the damage from lasers due to some common features with mammals (Martin, 2017). Yet, the differences between avian and mammalian eyes could influence how laser light is absorbed, reflected and transmitted and may affect the energy levels that could lead to retinal injury (Gabel and Birngruber, 1981; Chen et al., 2011; Tsukahara et al., 2014). For example, birds generally have smaller eyes and shorter focal lengths than non-human primates (Ross and Kirk, 2007; Hall and Heesy, 2011); thus, focusing light onto smaller areas of the retina could result in potentially higher energy densities impacting the retina, as was found in rabbits compared to humans (Gabel and Birngruber, 1981).

Given the degree to which birds rely on vision to find and consume food (Cazetta et al., 2009; Fernández-Juricic, 2012) and detect predators (Fernández-Juricic, 2012; Moore et al., 2013), eye injury or ocular diseases that harm visual function can have a negative impact on their overall fitness (Cousquer, 2005; Korbel, 2011). For instance, after ocular trauma, birds in wildlife rehabilitation centres position themselves so that the unaffected eye is directed towards objects of interest and make critical flight errors by missing perches or flying into walls (Pauli et al., 2007). Similarly, birds with cataracts are reluctant to fly, and even crash into objects when they do (Slatter et al., 1983), and exhibit weight loss and lethargy (Moore et al., 1985). Birds with an ocular *Mycoplasma sp.* infection stay at food patches for longer periods of time and decrease feeding efficiency compared to non-infected birds (Hotchkiss et al., 2005).

Due to the emergence of lasers as avian deterrents (Clausen et al., 2019; Brown and Brown, 2021), it is essential that we understand how laser eye exposure can affect the ability of animals to seek and consume food. Our goal was to assess how exposure to the laser as well as the energy level of that exposure would affect avian visual exploratory behaviour for the purpose of foraging as well as food consumption. This study is the first to assess the effects of laser on avian behaviour under controlled conditions that included a standardization in the exposure to the laser and the measurement of behaviour before and after exposure on each individual. We used house sparrows (*Passer domesticus*) as our study species. In a different study, we corroborated that house sparrows exposed to lasers developed different types of eye injuries (corneal oedema, cataracts, photoreceptor damage, etc.; Harris, 2021). Our overall a-priori hypothesis was that laser exposure could impair vision (see aforementioned mechanisms), which would modify the way birds visually seek and consume food. However, we did not have directional predictions for the different behavioural dimensions studied (see below) and consequently our study should be considered exploratory.

We studied fine-grained behavioural dimensions associated with visual exploratory behaviour in a foraging context that are explicitly related to the configuration of the visual system of our study species. House sparrows have laterally placed eyes that gives them wide visual coverage and an intermediate sized binocular field (30°), which is the result of the overlap between the right and left eyes fields (Fernández-Juricic et al., 2008). House sparrows also have a single centre of acute vision (i.e. fovea with the highest density of photoreceptors per unit area, increasing spatial resolving power) per eye (Dolan and Fernández-Juricic, 2010; Ensminger and Fernández-Juricic, 2014). Given the position of the fovea and the eyes, the projection of the centre of acute vision falls outside of the binocular field and into the lateral field (Ensminger and Fernández-Juricic, 2014). The implication is that house sparrows have relatively lower acuity vision when using their binocular field perpendicular to the foraging substrate, and to see the substrate with high visual resolution, they need to turn their heads sideways (either right or left) to use their foveal vision. Through rapid head movements, house sparrows explore the foraging substrate with a combination of binocular and foveal vision scans. We chose to study these different types of scans relative to laser exposure by first establishing baseline levels (before laser exposure) and then assessing changes within Week 1 and within Week 2 of laser exposure. Additionally, we manipulated the colour of the foraging substrate to generate two conditions that would make food items more or less visually contrasting to ascertain if animals with potential laser injuries would change their behaviour under challenging visual conditions.

## Materials and Methods

We captured a total of 40 house sparrows in West Lafayette and Lafayette, Indiana using potter traps from August 2017 to February 2018. We transferred all birds using soft bags to the indoor Purdue University animal care facility within 12 hours of capture, where we banded them, recorded sex and age, and randomly assigned them a laser exposure energy treatment (see below). Of the 40 house sparrows, 7 were adult females, 10 were juvenile females, 13 were adult males and 10 were juvenile males. We housed up to six birds in 61 × 61 × 76 cm mesh-wired enclosures under a 14-hour light/10-hour dark light cycle. We made water (with vitamins) available to them *ad libitum* and gave each bird a standard Petri dish (110 mm × 30 mm) with 80 g food mix per day. Food mix consisted of Purina game bird chow maintenance formula, black oils sunflower seeds, millet and dried meal worms. From capture until the animals were euthanized, we recorded regularly (often daily) their body mass. All housing conditions and protocols were approved by the Purdue Institutional Animal Care and Use Committee (protocol number 1707001594).

Our experiment consisted of five stages: (1) training, (2) before laser exposure trials, (3) laser exposure, (4) within Week 1 after laser exposure trials and (5) within Week 2 after laser exposure trials. All trials used the same experimental paradigm. We designed a food patch by modifying a standard Petri dish (110 mm × 30 mm) with a transparent plastic barrier wrapped around 2/3 of the dish perimeter (Appendix 1, Fig. A1.1). The barrier was made by cutting the plastic into triangular points to encourage birds to land on the unobstructed portion of the dish. We filled the dish with 32 g of plastic beads (TOHO, https://www.tohobeads.net/) so that the clear plastic bottom was not visible. Plastic beads were size 11/0, with hole size 0.7 mm, with the overall size being that of a single white millet seed. We then evenly dispersed 15 white millet seeds on top of the bead substrate. By manipulating the colour of the plastic bead substrate, we changed the contrast between the background substrate and the millet food items according to visual system of the house sparrow, whose parameters were measured in a previous study (Ensminger and Fernández-Juricic, 2014). More specifically, we calculated the chromatic and achromatic contrast of the millet seeds against different coloured plastic beads (visual background) to choose *high* and *low contrast* visual backgrounds (Appendix 1; Fig. A1.2). We chose to manipulate chromatic contrast due to the relative importance of chromatic cues versus achromatic cues when birds make foraging decisions (Stuart-Fox et al., 2004; Cazetta et al., 2009; Lind et al., 2013). Full details on each of the five stages are presented in Appendix 2. We provide a summary version in the next few paragraphs for the sake of space.

### Training

We trained birds how to forage for millet seeds in the same enclosures used for the trials by placing the training food patches with the millet and the beads from 1100 to 1115 and then evaluated the amount consumed (details in Appendix 2). We considered a bird trained when it consumed at least 50% of the seeds for the first time over a maximum of 2 days (one exposure per day).

### Before laser exposure trials

This treatment assessed baseline foraging behaviour before laser exposure. Trials were run in 0.61 × 0.61 × 0.76 m mesh-wired enclosures with one bird in each (details in Appendix 2; Fig. A2.1). Birds were food deprived overnight (food removed at 20:30) with trials beginning at 09:00 the next day. Each patch contained 15 millet seeds per trial. Each trial was either a high visual contrast (i.e. millet seeds were easy to resolve on the chromatic dimension) or a low visual contrast (i.e. millet seeds were more difficult to resolve on the chromatic dimension) based on the types of bead substrate used (Appendix 1). Each bird was randomly assigned to either the high or low seed visual contrast and completed one trial per day over two days, randomizing the presentation order. Trials lasted 15 minutes, after which we retrieved the food patches and counted the number of seeds left. After birds completed their trials, we weighed them and returned the regular food patches to the enclosures with 80 g of food mix.

### Laser exposure

Birds were administered a solution to dilate their pupils and then anaesthetized to eliminate small ocular movements that could alter the amount of laser light entering the eye and to reduce stress (details on the drugs and dosages in Appendix 2). We gently placed anaesthetized birds on a microwaved heating pad and several layers of towels in the laser exposure room (inspected and approved by Purdue Radiological and Environmental Management). We used a laser that was a prototype of the Seabird Saver (https://www.bmis-bycatch.org/mitigation-techniques/seabird-saver), had adjustable wattage (from 0 to 1000 mW), had a beam diameter of 4 cm at the aperture, beam divergence of 0.5 mrad, and 532 nm wavelength. We chose this laser unit because of its use in the fishing industry at the time of the study (https://www.bmis-bycatch.org/system/files/zotero_attachments/library_1/V2KTRUUX%20-%20SBWG6_Doc_23_Mustad_Autoline_Seabird_Saver_E_s_f.pdf). Although it would have been ideal to use a North American native marine species for our study, we could not justify its use ethically and logistically. The house sparrows is an introduced bird in North America, and their small size made them ideal to conduct our foraging behaviour experiments in the allocated research space.

The laser unit was taped securely on a table and fitted with a Thor labs 1-in optical beam shutter and shutter controller attached so the new laser aperture was 2.54 cm. Exactly 1 m from the laser aperture, we placed a power sensor (Ophir 30A-BB-18 power sensor). We visually aligned the centre of the power sensor with the laser beam by adjusting the height of the meter and moving the meter either left or right. When the reading from the power meter (Ophir Vega laser power meter) was the power desired for exposure, we marked the location of the centre of the meter, then moved the power meter approximately 6 cm directly backwards (details of the laser exposure setup in Appendix 3).

We strapped each bird into a foam cradle using Velcro straps and secured their feet. We placed the restrained bird on the marked location in front of the power meter exactly 1 m from the laser aperture such that one eye of the bird was centred with both the power sensor and laser beam (Appendix 3, Fig. A3.1). The eye facing the beam was temporarily secured open and exposed the bird to the appropriate power level and duration three times. We chose three exposures to replicate the likely conditions birds would experience the Seabird Saver unit when applied to bird deterrent control in fisheries (E.F. Melvin, personal observation). We waited 3 seconds between exposures (recommended by Bruce Stuck, Director of the Ocular Trauma Research Division at the US Army Institute of Surgical Research in San Antonio, Texas until 2013) to prevent possible additive effects (Thomsen, 1991; Lund and Sliney, 2014). We then repeated the same exposure procedure on the opposite eye. After both eyes were exposed, we removed the bird from the cradle and placed it back in the bag over a warmed pad to maintain its body temperature. We monitored birds until they were awake (between 30 minutes and 3 hours) and returned them to their individual cages with 80 g food and water *ad libitum*.

Each of the 40 birds used in the study was exposed to a single energy level. We consequently used 40 different energy levels to explore how a relatively broad range could influence foraging behaviour. Because no previous studies have determined the effects of laser exposure on bird behaviour, we based our range of energies on the accepted human laser safety guidelines (American National Standards Institute, 2014), which in turn stem from controlled experiments mostly in non-human primates (Farrer et al., 1970; Lund et al., 2007). In those experiments, the eye was exposed to incremental dosages and assessed for signs of damage (Zwick et al., 1994; Lund et al., 2007). Based on the American National Standard for Safe Use of Lasers and the International Commission on Non-Ionizing Radiation Protection (American National Standards Institute, 2014), the threshold for laser damage is the dose at which an individual has a 50% probability of having damage. We used the predicted ED50 values as median doses and exposed birds to values three times below the lowest predicted ED50 and three times above the highest predicted ED50. The rationale behind this strategy was to have a wide range of energy values to assess the behavioural consequences of laser exposure. First, we calculated the maximum permissible exposure (MPE), which is one tenth of the ED50 (American National Standards Institute, 2014). Like the ED50, the MPE can be expressed as the radiant energy per unit area in $\frac{mJ}{c{m}^2}$, also called corneal irradiance (American National Standards Institute, 2014). We used the following equation to calculate the MPE of continuous wave lasers (like our unit) that are 400 to 700 nm for laser exposure times between 5 μs and 10 s:


$$ \mathrm{MPE}=1.8\ast{t}^{0.75}; $$

where *t* is laser exposure time in s (American National Standards Institute, 2014). We chose seven laser exposure times (0.1, 0.25, 0.4, 0.55, 0.7, 0.85, 1.0 seconds) based on times found in the literature that we could attain with our equipment (Ham et al., 1970; Leibu et al., 1999).

The equations used are based on a human pupil diameter of 7 mm, so we corrected them for a 2.22-mm house sparrow pupil. We corrected the MPE values by multiplying them by the ratio (approximately 5.52) of human pupil area (38.48 mm2) to house sparrow pupil area (6.97 mm^2^, measured on a dilated eye). This gave us a range of MPE values from 1.77 mJ/cm^2^ to 9.94 mJ/cm^2^, which we then multiplied by 10 to get the predicted ED50s. We divided the ED50s by three because we planned to expose each eye three times. The final predicted ED50 values, which represented our predicted threshold of laser eye injury for house sparrows when exposed to a laser three times for 0.1 to 1 second, ranged from 5.89 to 33.12 mJ/cm^2^. We then estimated the corneal irradiances that were approximately three times below the lowest predicted ED50 and three times above the highest predicted ED50 to obtain our final irradiance values. Our range of corneal irradiances were 1.96 to 99.36 mJ/cm^2^. The next step was to apply the final irradiances using the laser, which required the manipulation of two factors: laser power and laser exposure time. We used the following mathematical relationship that we modified from the ANSI guidelines (American National Standards Institute, 2014):


\begin{align*} &\left(\frac{\mathrm{Power}\ mW}{\mathrm{Beam}\ \mathrm{Area}\ \mathrm{at}\ \mathrm{Cornea}\ {cm}^2}\right)\ast \mathrm{time}\ s\\&\quad =\mathrm{Corneal}\ \mathrm{Irradiance}\frac{\mathrm{mJ}}{{\mathrm{cm}}^2}; \end{align*}

where the beam area at cornea was 5.07 cm^2^, based on the 2.54-cm diameter of the shutter we fitted to the laser. We chose 7 laser powers (60, 90, 130, 165, 200, 235, 270 mW) to substitute into this equation that were low compared to our possible range of powers (0–1000 mW) and would give us final irradiances that were within the range we wanted. We assumed beam size did not change from aperture to the cornea because the beam divergence was low (0.5 mrad). Using the 7 powers and times mentioned earlier, we calculated 49 different irradiances ranging from 1.15 to 53.2 mJ/cm^2^. We then converted them to total intraocular energies (TIEs) by multiplying the values by the area of a 2.22 mm house sparrow pupil, which gave values in mJ. The final energies for the house sparrows ranged from 0.08 to 3.71 mJ (0.08, 0.13, 0.18, 0.21, 0.23, 0.27, 0.32, 0.33, 0.37, 0.45, 0.52, 0.57, 0.58, 0.69, 0.70, 0.71, 0.72, 0.81, 0.82, 0.91, 0.93, 1.10, 1.11, 1.25, 1.31, 1.48, 1.51, 1.59, 1.78, 1.79, 1.92, 1.93, 2.04, 2.26, 2.27, 2.34, 2.60, 2.75, 3.16, 3.23, 3.71 mJ).

### After laser exposure trials

The birds participated in the after-exposure trial following the same procedure as the before laser-exposure trials approximately 24 hours after laser exposure, and again the day after that, approximately 48 hours after exposure (two trials to expose them to the low and high food visual contrast conditions presented randomly). We called these two trials ‘within Week 1’ trials. Seven and eight days after the laser exposure, birds participated in another set of two after-exposure trials (with low and high food visual contrast conditions presented randomly). We called these two trials ‘within Week 2’ trials. Due to an error in planning for within Week 2 trials, three out of the 40 birds had their second after-exposure trial on the tenth day after laser exposure. After the within Week 2 trials, birds were weighed and euthanized with CO_2_.

### Behavioural analysis

**Figure 1:**
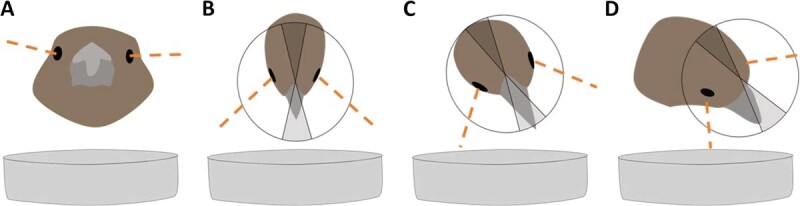
Head positions recorded during the behavioural trials. Dashed lines indicate the foveal projections for the right and left eyes, and the grey portions of the visual field in front of the bill indicate the binocular visual field width of house sparrows. (a) Scanning position with the bill above or slightly below the horizontal plane but not projecting into the food patch. (b) Individual exploring the food patch with binocular vision, with the bill projecting into the patch and the head not tilted. (c) Individual using a combination of binocular–foveal vision, with the bill and one fovea (either right or left) projecting into the food patch. (d) Individual using foveal vision, with one fovea (either right or left) but not the bill projecting into the food patch.

We compiled the videos from the six foraging trials for each bird that participated in the three time conditions (before laser exposure, within Week 1 after laser exposure, within Week 2 after exposure) and two food visual contrast conditions (high contrast, low contrast). Using the program BORIS (Friard and Gamba, 2016), we developed an ethogram to analyse fine-grained visual exploratory behaviour of the food patch and used the frame-by-frame function to record behaviour every 0.033 seconds for 30 seconds (see full list of behaviours coded in Appendix 4). The 30 seconds started from the moment the bird arrived at the food patch (both feet in contact with the platform or dish) and only included the time the bird remained at the food patch. During the 30 seconds, we recorded pecks (beak moves towards the substrate and makes contact with it), seeds eaten (seed seen in the bird beak, chewing and possibly husk flying), changes in head position and when the bird left the food patch (both feet no longer in contact with platform or dish). We focused on the first 30 seconds of the interactions with the foraging substrate to capture the initial instances of the animal exploring the substrate with its eyes to minimize the occurrence of compensatory behaviour (due to an eye injury) or habituation to the initial spatial position of the seeds over the trial time. We recorded the bird's head position at the first clear frame after a bird had changed head position and classified the position relative to the foraging substrate as: head-up scanning, using binocular vision to explore the substrate, using foveal vision to explore the substrate or using both binocular and foveal vision to explore the substrate. We were able to classify head positions based on the portion of the visual field used following previous work measuring these traits in house sparrows (Fernández-Juricic et al., 2008; Dolan and Fernández-Juricic, 2010; Ensminger and Fernández-Juricic, 2014). We considered the individual was head-up scanning (Fig. 1a) when the beak was below the horizontal plane but not projecting into the food patch or the beak was above the horizontal plane. We considered the individual was using foveal vision when the head was tilted sideways (either right or left) and the fovea of one eye (i.e. centre of acute vision) was projecting into the food patch (Fig. 1d). We considered the individual was using its binocular–foveal vision when the head was slightly sideways such that both foveal and binocular vision were potentially projecting into the foraging substrate (Fig. 1c). We did not assume that the binocular–foveal vision head position meant that the animal was necessarily using both fields of view as centres of attention, as our level of resolution could not establish what was the source of visual attention at that head position. We considered the individual was using binocular vision when the binocular field (i.e. overlap in the visual field of the right and left eyes) was projecting into the food patch (i.e. beak perpendicular to foraging substrate) and the head was not tilted (Fig. 1b). In the foveal or binocular–foveal vision positions, we also coded whether the animal was using the right or left eye given the body of evidence that birds can use either eye differently depending on the task (Franklin and Lima, 2001; Templeton and Gonzalez, 2004; Butler et al., 2018).

After we scored the videos, we used BORIS time–budget analysis tool to export the data to Microsoft Excel in individual files, one per trial per bird. Each data file contained the number of times a behaviour occurred and the duration of each behaviour, from which we estimated the percentage of times and rates (events per min) of occurrence of different behaviours. Given the very short durations of head-up scans, foveal visual explorations, seed pecks and seed consumption events, we only analysed their rates. Given the relatively longer duration of binocular visual explorations, we only analysed the percentage of time spent in that head-position. We analysed both the rates and time percentage of foveal–binocular vision given the mixed nature of this head-position. We also recorded the total number of seeds birds consumed after the 15-minute trial to calculate giving-up density (i.e. number of seeds eaten after the total 15-minute trial). Finally, we recorded the time period from the beginning of a trial until the individual first arrived at the food patch as latency to visit the food patch (seconds).

### Statistical analysis

We divided our statistical analyses into two sections: laser exposure effects and laser energy effects. In the laser exposure effects section, we focused on how different behavioural responses of house sparrows varied after laser exposure as well as between the two time points after laser exposure (within Week 1, within Week 2) compared to before laser exposure, also considering the effects of food visual contrast. In this first section, we did not consider the effects of different laser energy levels because in the before laser exposure treatment, individuals had no exposure to any laser energy level by design. In the laser energy effects section, we were interested in assessing the effects of energy levels relative to the other factors studied (considering food visual contrast when appropriate) by leaving out the before laser exposure treatment and only including both after laser exposure treatments (within Week 1, within Week 2).

In both sections, we used general linear mixed models ran with the R package afex (Singmann et al., 2023). We analysed the following dependent variables: latency to visit food patch (sec), percent of time using binocular vision, percent of time using binocular–foveal vision, binocular–foveal vision rate (events per min), foveal vision rate (events per min), pecking rate (events per min), seed consumption rate (events per min), seed giving-up density (i.e. number of seeds left at the end of the trial), scanning rate (events per min). We checked for the homogeneity of variance and normality of the error assumptions; most of the models met these assumptions, but a couple of them had minor deviations. We decided not to transform the data to facilitate the detection of interaction effects, which may be masked with some transformations (Belzak and Bauer, 2019). In all models, individual was included as a random factor and trial order as a fixed effect to control for the potential confounding factor of individuals modifying their behaviour over multiple treatment exposures. In the laser exposure effects section, for most of the dependent variables, we considered two within-subject factors: laser exposure (before, within Week 1 after, within Week 2 after) and seed visual contrast (low, high) and their interaction. For percent of time using binocular–foveal vision, binocular–foveal vision rate and foveal vision rate, we considered a third within-subject factor (visual field; right, left), and all interactions between factors, to assess potential visual laterality effects when inspecting the food patch. Following Singmann and Kellen (2019), we assessed random structures with different levels of complexity (from more to less complex) until the model would converge. From this process, we chose the following random structures for models with two and three within-subject factors: (within-subject factor *a* + within-subject factor *b* || *bird id*; with random intercepts and random slopes but excluding the correlations between intercepts and slopes) and (within-subject factor a + within-subject factor b + within-subject factor c || bird id; with random intercepts and random slopes but excluding the correlations between intercepts and slopes), respectively.

In the laser energy effects section, for most of the dependent variables, we considered two within-subject factors (laser exposure (within Week 1 after, within Week 2 after)) and seed visual contrast (low, high) along with laser energy (continuous) and their interactions. We chose the same two within-subject factor random structures as described above. For percent of time using binocular–foveal vision, binocular–foveal vision rate and foveal vision rate, the consideration of a third within-subject factor (visual field; right, left) along with the continuous factor energy level and all their interactions prevented these complex models from converging. Therefore, for these three behavioural responses, we only included the within-subject factors that turned out to be significant in the laser exposure effects section (see Results), laser energy and their interactions. Two of these models had a single within-subject factor, leading to the following random structure: (within-subject factor a || bird id; with random intercepts and random slopes but excluding the correlations between the intercept and slopes).

Finally, we analysed body mass changes before and after laser exposure by running a general linear mixed model with laser exposure (before, after), sex (male, female), age (juvenile, adult) and days since first body mass measure (within each before and after laser exposure period) as independent factors. We also included in the model the interactions between laser exposure and (a) sex, (b) age and (c) days since first body mass measure. Bird id was considered a random effect and laser exposure a within-subject factor, leading to the following random structure: (within-subject factor | bird id; with random intercepts and random slopes). Days since the first body mass measure was centred around 0 in the mixed model. For this analysis, we could not collect data from one of our focal birds due to logistical reasons.

We used R package emmeans (Lenth et al., 2019) to estimate the means and SE’s for different treatment values. We also used R package afex (Singmann et al., 2023) to plot the effects of one or two within-subject factors using the function afex_plot, which considered the random bird id effects for the estimation of the means and SEs. We used the R package interactions (Long, 2019) to plot the interactions between categorical and continuous factors using the function interact_plot, which portrayed the predicted lines with 95% confidence bands.

## Results

### Laser exposure effects

**Figure 2:**
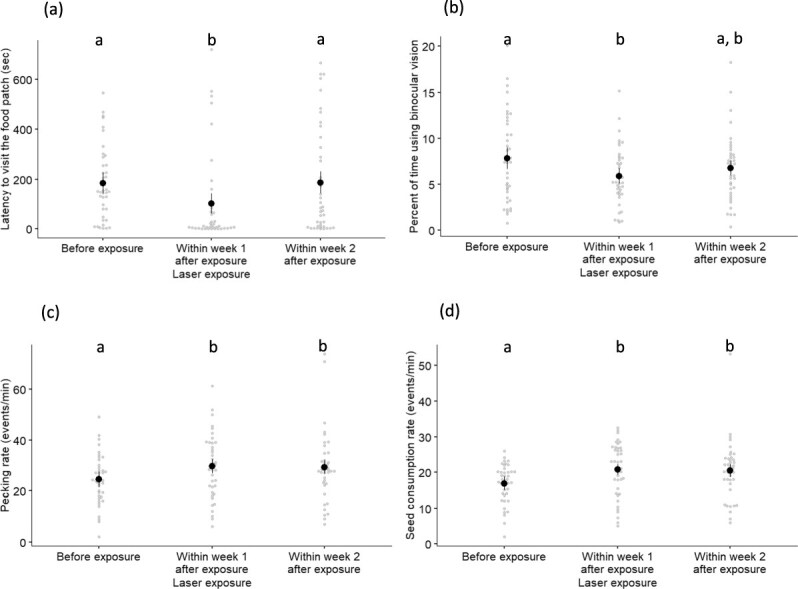
Effects of laser exposure on house sparrow behaviour. (a) Latency to visit the food patch, (b) percentage of time using binocular vision looking at the food patch, (c) pecking rate, and (d) seed consumption rate relative to the timing of laser exposure (before, within Week 1 after exposure, within Week 2 after exposure). Shown are means, SEs, and raw data points. Letters indicate the results of pairwise comparisons (see text for details).

The latency of house sparrows to visit the food patch right after the trials began was significantly affected by laser exposure (Table 1; Fig. 2a), but we did not detect a significant effect of seed visual contrast, trial order or its interaction with laser exposure and trial order (Table 1). Individuals approached the food patch faster within Week 1 after exposure than they did before laser exposure (*z* ratio = 2.89, *P* = 0.011; Fig. 2a) and faster than within Week 2 after exposure (*z* ratio = −2.83, *P* = 0.013; Fig. 2a). There was no significant difference in latency between before and within Week 2 after laser exposure (z ratio = −0.06, *P* = 0.998; Fig. 2a).

**Table 1:** Effects of laser exposure (before, within Week 1 after, within Week 2 after), seed visual contrast (high, low), visual field (right, left) and their interactions on different house sparrow behavioural responses related to using different areas their visual system relative to the food patch (binocular vision, foveal vision), pecking, scanning, and the latency to visit the food patch. Results from general linear mixed models (significant values are in bold emphasis)

	***F***	***d.f***	***P***
**Latency to visit food patch (sec)**			
Laser exposure	5.69	2, 51.1	**0.006**
Seed visual contrast	0.33	1, 38.64	0.569
Trial order	2.94	1, 127.3	0.089
Laser exposure X Seed visual contrast	0.06	2, 89.7	0.940
**Percent of time using binocular vision**			
Laser exposure	3.78	2, 51.1	**0.030**
Seed visual contrast	0.00	1, 38.7	0.980
Trial order	0.06	1, 130.2	0.815
Laser exposure X Seed visual contrast	0.43	2, 91.9	0.651
**Percent of time using binocular–foveal vision**			
Laser exposure	0.44	2, 52.4	0.644
Seed visual contrast	0.06	1, 38.6	0.802
Visual field	10.13	1, 39	**0.003**
Trial order	8.09	1, 194.5	**0.004**
Laser exposure X Seed visual contrast	0.74	2, 281.2	0.479
Laser exposure X Visual field	0.68	2, 279.6	0.507
Seed visual contrast X Visual field	0.07	2, 279.6	0.792
Laser exposure X Seed visual contrast X Visual field	0.13	2, 279.6	0.874
**Binocular–foveal vision rate (events per min)**			
Laser exposure	0.65	2, 52.3	0.526
Seed visual contrast	0.23	1, 38.6	0.636
Visual field	13.02	1, 39.00	**0.001**
Trial order	1.05	1, 194.4	0.308
Laser exposure X Seed visual contrast	0.83	2, 280.5	0.437
Laser exposure X Visual field	2.11	2, 278.8	0.122
Seed visual contrast X Visual field	0.55	1, 278.8	0.457
Laser exposure X Seed visual contrast X Visual field	1.55	2, 278.8	0.214
**Foveal vision rate (events/min)**			
Laser exposure	0.28	2, 52.9	0.757
Seed visual contrast	0.73	1, 38.6	0.400
Visual field	22.57	1, 39.0	**< 0.001**
Trial order	0.61	1, 223.2	0.434
Laser exposure X Seed visual contrast	0.08	2, 284.1	0.919
Laser exposure X Visual field	6.96	2, 283.1	**0.001**
Seed visual contrast X Visual field	0.53	1, 283.1	0.469
Laser exposure X Seed visual contrast X Visual field	0.16	2, 283.1	0.851

(Continued)

**Table 1:** Continued

	***F***	***d.f***	***P***
**Pecking rate (events/min)**			
Laser exposure	4.77	2, 51.2	**0.013**
Seed visual contrast	1.57	1, 38.6	0.218
Trial order	0.11	1, 122.2	0.742
Laser exposure X Seed visual contrast	1.79	2, 86.9	0.172
**Seed consumption rate (events/min)**			
Laser exposure	6.07	2, 51.1	**0.004**
Seed visual contrast	6.77	1, 38.6	**0.013**
Trial order	0.98	1, 123.5	0.325
Laser exposure X Seed visual contrast	1.08	2, 88.6	0.343
**Seed giving-up density**			
Laser exposure	2.14	2, 51.2	0.128
Seed visual contrast	2.68	1, 38.6	0.109
Trial order	1.19	1, 123.6	0.278
Laser exposure X Seed visual contrast	2.13	2, 88.8	0.124
**Scanning rate (events/min)**			
Laser exposure	1.35	2, 51.2	0.269
Seed visual contrast	8.52	1, 38.7	**0.006**
Trial order	0.16	1, 124.8	0.689
Laser exposure X Seed visual contrast	4.07	2, 86.3	**0.020**

House sparrows changed the percentage of time using binocular vision when exploring the food patch relative to laser exposure (Table 1, Fig. 2b). The percentage of time allocated to binocular vision decreased significantly within Week 1 after laser exposure compared to before exposure (z ratio = 1.94, *P* = 0.016; Fig. 2b); however, no significant differences were detected between within Week 2 after exposure compared to before exposure (*z* ratio = 1.06, *P* = 0.323; Fig. 2b) or between within Week 1 and within Week 2 after exposure (z ratio = −0.88, *P* = 0.422). Seed visual contrast and its interaction with laser exposure, along with trial order, were not significant (Table 1).

Both the percentage of time house sparrows allocated to looking at the food patch with binocular–foveal vision and the rate at which they used binocular–foveal vision when looking at the food patch were both influenced significantly by visual field (i.e. right or left eye) (Table 1). Individuals spent more time (right visual field, 4.95 ± 0.50% of total time by food patch; left visual field, 7.33 ± 0.50% of total time by food patch) and looked more often (right visual field, 10.20 ± 0.95 events/min; left visual field, 15.50 ± 0.95 events/min) with the left than the right visual field, irrespective of laser exposure and seed visual contrast (Table 1). Additionally, trial order significantly affected the percentage of time using binocular–foveal vision (Table 1), which was higher in the first (6.65 ± 0.37%) than in the second (5.63 ± 0.37%) trial within a given laser exposure treatment (before, within Week 1, within Week 2).

**Figure 3:**
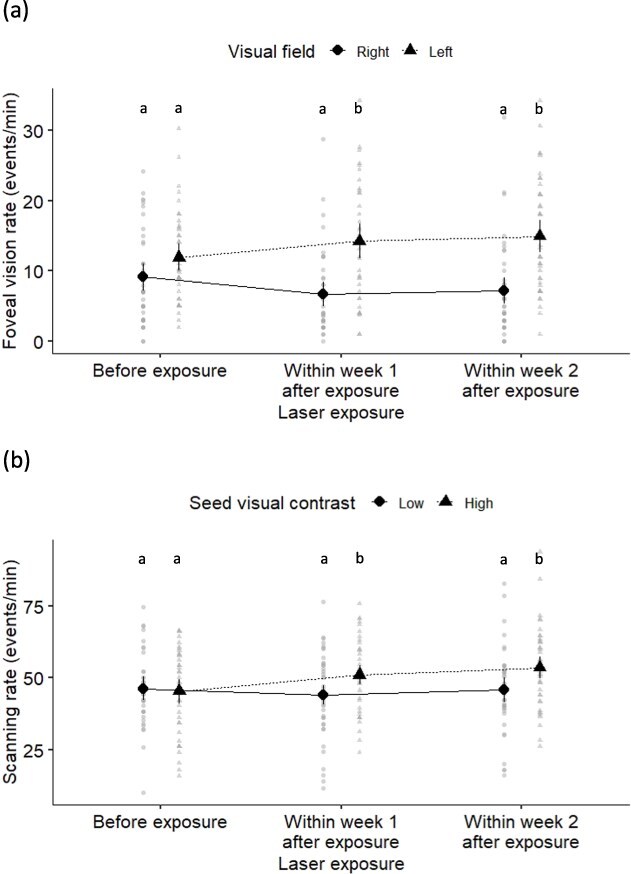
Effects of laser exposure on house sparrow behaviour. (a) Rate of use of foveal vision towards the food patch relative to the timing of laser exposure (before, within Week 1 after exposure, within Week 2 after exposure) and the visual field (right, left). (b) Rate of scanning the environment relative to the timing of laser exposure and the visual contrasts of seeds (low, high). Letters indicate the results of pairwise comparisons (see text for details).

The rate at which house sparrows looked at the food patch with foveal vision was significantly affected by the visual field (right eye, left eye) as well as the interaction between laser exposure and visual field (Table 1). Overall, individuals used left foveal vision (13.69 ± 0.88 events/min) more often than right foveal vision (7.69 ± 0.88 events/min). Interestingly, this visual field effect was a function of laser exposure (Fig. 3a); such that before laser exposure there was not significant difference in foveal vision rate between right and left visual fields (*z* ratio = −1.82, *P* = 0.070), but the left eye was used more for foveal visual exploratory behaviour than the right eye within Week 1 (*z* ratio = −4.92, *P* < 0.001) and within Week 2 (*z* ratio = −5.03, *P* < 0.001) after laser exposure (Fig. 3a).

The rate at which house sparrows pecked at seeds was affected significantly by laser exposure (Table 1; Fig. 2c), but we did not detect a significant effect of seed visual contrast or its interaction with laser exposure (Table 1). Individuals had higher peck rates within Week 1 after laser exposure than before laser exposure (*z* ratio = −2.97, *P* = 0.008) as well as within Week 2 after laser exposure than before laser exposure (*z* ratio = −2.35, *P* = 0.049; Fig. 2c). We did not find a significant difference in peck rate between within Week 1 and within Week 2 after laser exposure (*z* ratio = 0.15, *P* = 0.988; Fig. 2c).

The rate at which house sparrows consumed seeds at the beginning of the trial was significantly affected by both laser exposure and seed visual contrast, but not by their interaction (Table 1). House sparrows consumed more seeds per min within Week 1 after laser exposure than before laser exposure (*z* ratio = −3.40, *P* = 0.002) and within Week 2 after laser exposure than before laser exposure (*z* ratio = −2.69, *P* = 0.020; Fig. 2d). We did not find a significant difference in seed consumption rate between within Week 1 and within Week 2 after laser exposure (*z* ratio = 0.20, *P* = 0.978; Fig. 2d). Additionally, house sparrows consumed more seeds per min in the high (20.5 ± 1.0 events/min) than in the low (18.3 ± 1.0 events/min) seed visual contrast treatment (Table 1). Despite this variation in seed consumption rate within 30 seconds of landing in the food patch, seed giving-up densities including the overall trial time (~ 15 minutes) did not vary significantly with any of the studied factors (Table 1).

House sparrows scanning rate was affected significantly by both seed visual contrast and the interaction between laser exposure and seed visual contrast (Table 1). Individuals scanned at a lower rate when seeds were more visually challenging to perceive (i.e. low visual contrast, 45.30 ± 1.60 events/min) than when they were more easily distinguished from the visual background (i.e. high visual contrast, 50.0 ± 1.6 events/min). However, this effect was a function of laser treatment due to the significant interaction effect (Fig. 3b). There was no significant difference in scanning rate between seed visual contrast treatments before laser exposure (*z* ratio = 0.33, *P* = 0.742), but the higher scanning rate in high compared to low seed visual contrast conditions occurred within Week 1 (*z* ratio = −2.82, *P* = 0.005) and within Week 2 (*z* ratio = −3.11, *P* = 0.002) after laser exposure (Fig. 3b).

### Laser energy effects

**Figure 4:**
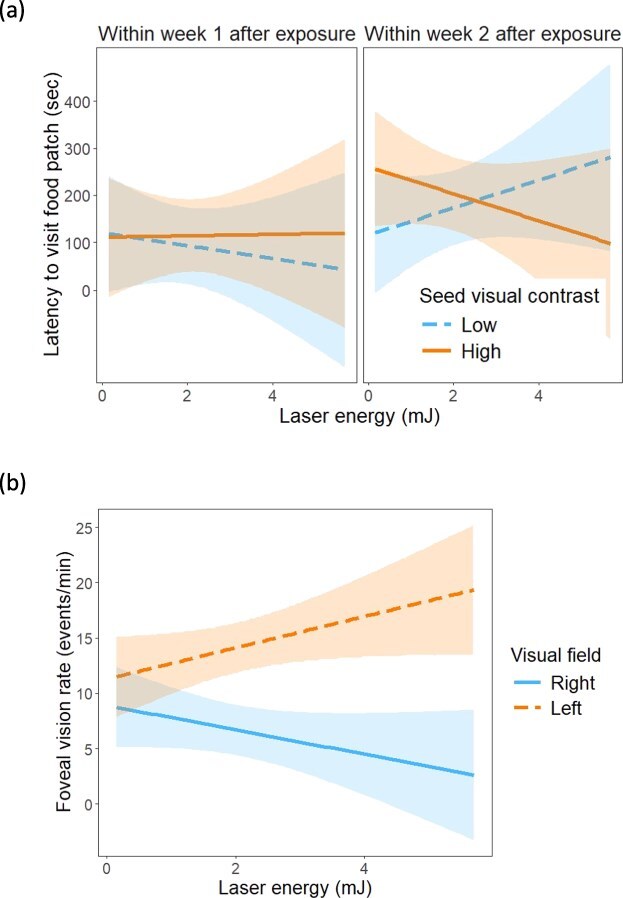
Effects of laser energy on house sparrow behaviour considering the treatments after laser exposure. (a) Latency to visit the food patch relative to laser exposure (within Week 1 after, within Week 2 after), seed visual contrast (low, high), and laser energy level. (b) Rate of use of foveal vision towards the food patch relative to the visual field (right, left) and laser energy level.

After house sparrows were exposed to the laser, their latency to visit the food patch was significantly affected by the three-way interaction among laser exposure, contrast and laser energy (Table 2, Fig. 4a). Within Week 1 after laser exposure, the latency to visit the food patch did not seem to vary with energy at high seed visual contrast; however, at low seed visual contrast (i.e. seeds were more difficult to resolve), animals that had been exposed to laser energies higher than 1 mJ tended to arrive sooner to the food patches (Fig. 4a). However, this pattern changed within Week 2 after laser exposure, such that at low seed visual contrast (i.e. seeds were more difficult to detect), latency to visit the food patch increased with higher laser energy exposure, whereas at high seed visual contrast (i.e. seeds were easier to resolve), latency decreased with higher laser energy exposure (Fig. 4a). Interestingly, below 2.5 mJ, latency was higher in the high vs. low visual contrast treatments, but above this threshold, the pattern reversed (Fig. 4a).

**Table 2:** Effects of laser exposure (within Week 1 after, within Week 2 after), seed visual contrast (high, low), laser energy, visual field (right, left) and their interactions on different house sparrow behavioural responses related to using different areas their visual system relative to the food patch (binocular vision, foveal vision), pecking, scanning, and the latency to visit the food patch. Results from general linear mixed models (significant values are bold in emphasis)

	***F***	***d.f***	***p***
**Latency to visit food patch (sec)**			
Laser exposure	1.89	1, 38.0	0.177
Seed visual contrast	1.62	1, 37.4	0.211
Laser energy	0.02	1, 38.0	0.882
Trial order	0.04	1, 68.2	0.852
Laser exposure X Seed visual contrast	4.09	1, 38.2	0.051
Laser exposure X Laser energy	0.09	1, 38.0	0.762
Seed visual contrast X Laser energy	1.00	1, 37.8	0.324
Laser exposure X Seed visual contrast X Laser energy	5.48	1, 39.3	**0.024**
**Percent of time using binocular vision**			
Laser exposure	2.67	1, 38.0	0.203
Seed visual contrast	0.12	1, 37.2	0.667
Laser energy	0.17	1, 38.0	0.682
Trial order	0.00	1, 74.9	0.950
Laser exposure X Seed visual contrast	0.06	1, 38.4	0.808
Laser exposure X Laser energy	0.14	1, 38.0	0.708
Seed visual contrast X Laser energy	0.18	1, 37.7	0.896
Laser exposure X Seed visual contrast X Laser energy	0.04	1, 39.7	0.850
**Percent of time using binocular–foveal vision**			
Visual field	1.96	1, 38	0.169
Laser energy	0.13	1, 38	0.717
Trial order	0.76	1, 239	0.385
Visual field X Laser energy	0.26	1, 38	0.616
**Binocular–foveal vision rate (events/min)**			
Visual field	2.12	1, 38	0.153
Laser energy	0.10	1, 38	0.757
Trial order	0.09	1, 239	0.758
Visual field X Laser energy	0.59	1, 38	0.447
**Foveal vision rate (events/min)**			
Laser exposure	0.49	1, 38	0.486
Visual field	0.49	1, 38	0.487
Laser energy	1.15	1, 38	0.290
Trial order	0.02	1, 197	0.875
Laser exposure X Visual field	0.02	1, 197	0.569
Laser exposure X Laser energy	2.18	1, 38	0.148
Visual field X Laser energy	9.30	1, 38	**0.004**
Laser exposure X Visual field X Laser energy	0.62	1, 197	0.430

(Continued)

**Table 2:** Continued

	***F***	***d.f***	***p***
**Peck rate (events/min)**			
Laser exposure	0.06	1, 38.0	0.816
Seed visual contrast	3.07	1, 37.2	0.088
Laser energy	0.48	1, 38.0	0.492
Trial order	0.03	1, 74.9	0.872
Laser exposure X Seed visual contrast	4.38	1, 38.5	**0.043**
Laser exposure X Laser energy	0.03	1, 38.0	0.859
Seed visual contrast X Laser energy	0.33	1, 37.6	0.570
Laser exposure X Seed visual contrast X Laser energy	6.98	1, 39.7	**0.012**
**Seed consumption rate (events/min)**			
Laser exposure	0.09	1, 38.0	0.765
Seed visual contrast	4.54	1, 37.2	**0.039**
Laser energy	0.16	1, 38.0	0.687
Trial order	0.40	1, 74.9	0.528
Laser exposure X Seed visual contrast	5.02	1, 37.6	**0.031**
Laser exposure X Laser energy	0.05	1, 38.0	0.822
Seed visual contrast X Laser energy	0.20	1, 37.6	0.658
Laser exposure X Seed visual contrast X Laser energy	7.20	1, 39.7	**0.011**
**Seed giving-up density**			
Laser exposure	4.25	1, 38	**0.046**
Seed visual contrast	2.40	1, 37.2	0.130
Laser energy	0.17	1, 38	0.685
Trial order	3.89	1, 74.9	0.052
Laser exposure X Seed visual contrast	0.26	1, 38.5	0.613
Laser exposure X Laser energy	1.89	1, 38	0.177
Seed visual contrast X Laser energy	1.45	1, 37.6	0.237
Laser exposure X Seed visual contrast X Laser energy	0.32	1, 39.7	0.573
**Scanning rate (events/min)**			
Laser exposure	0.01	1, 38.0	0.908
Seed visual contrast	9.10	1, 37.2	**0.005**
Laser energy	0.01	1, 38.0	0.914
Trial order	0.11	1, 74.9	0.743
Laser exposure X Seed visual contrast	0.24	1, 38.5	0.630
Laser exposure X Laser energy	0.90	1, 38.0	0.347
Seed visual contrast X Laser energy	0.39	1, 37.6	0.534
Laser exposure X Seed visual contrast X Laser energy	0.18	1, 39.7	0.678

After laser exposure, the percentage of time house sparrows spent looking at the food patch with their binocular vision and binocular–foveal vision, as well as the rate of binocular- foveal vision, were not affected significantly by laser energy or any of the other factors considered (Table 2).

After laser exposure, the rate house sparrows looked at the food patch with foveal vision was significantly affected by the interaction between visual field and laser energy (Table 2). At laser energies higher than 1 mJ, the use of left foveal vision increased, and right foveal vision decreased, with an increase in the energy of the laser (Fig. 4b).

**Figure 5:**
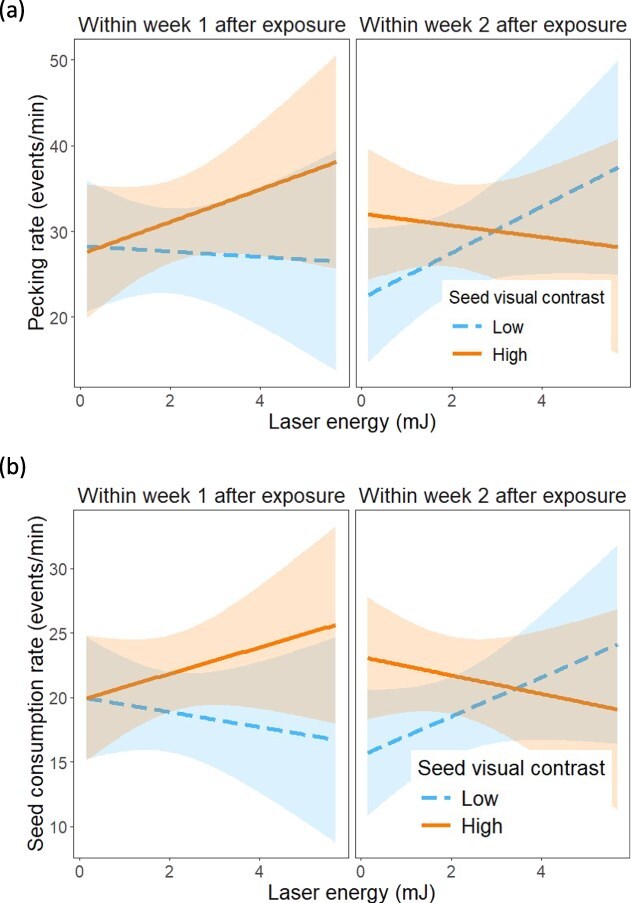
Effects of laser energy on house sparrow behaviour considering the treatments after laser exposure. (a) Seed pecking rate and (b) seed consumption rate relative to laser exposure (within Week 1 after, within Week 2 after), seed visual contrast (low, high) and laser energy level. Shown are predicted trends by the model with 95% confidence bands.

After laser exposure, seed pecking rate was affected significantly by the interaction between laser exposure and seed visual contrast, but also by the three-way interaction among laser exposure, seed visual contrast and laser energy (Table 2). Within Week 1 after exposure, the trend towards higher pecking rates in the high seed visual contrast treatment was more pronounced for those individuals that had been exposed to laser energy levels higher than 1 mJ (Fig. 5a). Within Week 2 after exposure, pecking rates in the low seed visual contrast treatment increased with energy, and the opposite pattern was found under high visual contrast, such that pecking rates were higher in the high vs. low visual contrast below 3 mJ, and lower in the high vs. low visual contrast above this threshold energy (Fig. 5a).

Furthermore, after laser exposure, seed consumption rate was affected significantly by seed visual contrast, the two-way interaction between laser exposure and contrast and the three-way interaction among laser exposure, seed visual contrast and laser energy (Table 2). House sparrows had higher seed consumption rates when the seeds were more visually contrasting (22.10 ± 1.23 events/min) than when they were less visually contrasting (19.10 ± 1.23 events/min). However, this effect was a function of the timing of laser exposure and its energy levels (Fig. 5b). Within Week 1 after exposure, at energy levels higher than 1 mJ, seed consumption rates increased with energy in the high seed visual contrast treatment, but decreased with energy in the low visual contrast treatment (Fig. 5b). Within Week 2 after exposure, individuals increased seed consumption rates with laser energy under low visual contrast, and the reversed pattern was found under high visual contrast (Fig. 5b). At laser energy levels below 3.75 mJ, seed consumption rate was overall higher in the high than in the low visual contrast treatment, but above 3.75 mJ, we found the reversed pattern (Fig. 5b). Notwithstanding the variation in seed consumption rates within 30 seconds of landing in the food patch, seed giving-up densities over the whole trial time (~ 15 minutes) varied significantly only with laser exposure (higher within Week 2 (1.2 ± 0.3) than within Week 1 (0.56 ± 0.3)), but not with laser energy (Table 2).

After animals were exposed to the laser, their scanning rates were only significantly affected by seed visual contrast (Table 2), as reported before, but not by laser energy. House sparrows scanned more under the high seed visual contrast (52.2 ± 1.9 events/min) than the low seed visual contrast (44.9 ± 1.9 events/min) treatments.

### Body mass changes

**Figure 6:**
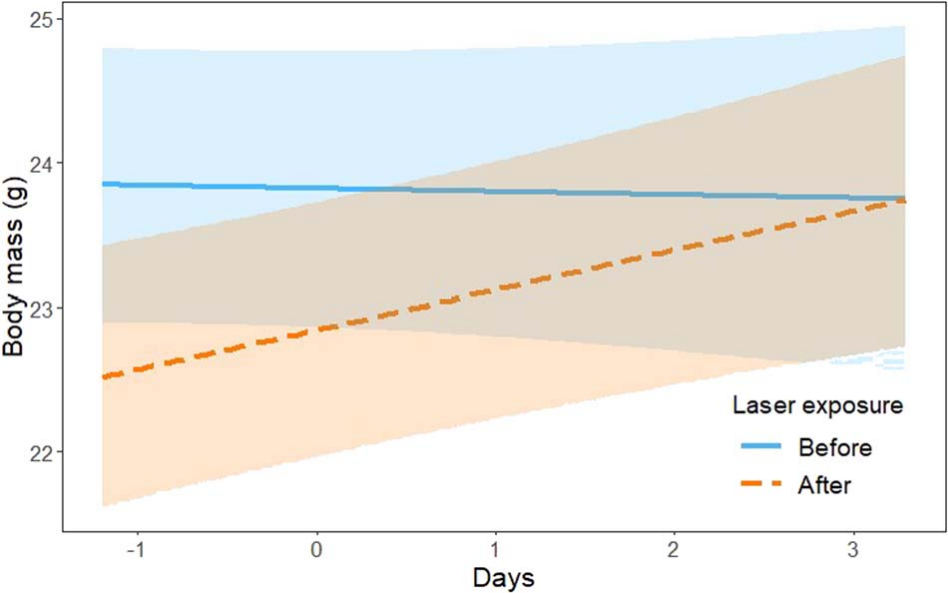
Effects of days since first body weight measurement (centred around 0) on house sparrow body mass before and after energy exposure. Shown are predicted trends by the model with 95% confidence bands.

Body mass was significantly affected by laser exposure (*F*_1, 40.3_ = 14.43, *P* < 0.001), sex (*F*_1, 35.9_ = 4.85, *P* = 0.034), age (*F*_1, 36.6_ = 13.3, *P* < 0.001), and days since first measurement (*F*_1, 407.3_ = 3.94, *P* = 0.047), with no significant effects of the interaction between laser exposure and sex (*F*_1, 34.4_ = 1.29, *P* = 0.263), and laser exposure and age (*F*_1, 44.4_ = 3.41, *P* = 0.071). Overall, body mass was lower after laser exposure (before, 24.90 ± 0.24; after, 24.40 ± 0.23), higher for males (males, 25.20 ± 0.30; females, 24.20 ± 0.34), higher for juveniles (juveniles, 25.50 ± 0.30; adults, 23.80 ± 0.34) and increased over time (slope = 0.13 ± 0.06). However, we found a significant interaction between laser exposure and days since first measurement (*F*_1, 403.4_ = 5.50, *P* = 0.019), whereby body mass did not change substantially in the days before laser exposure, but immediately after laser exposure, there was a marked decrease in body mass, followed by an increase up to the body weight before laser exposure (Fig. 6).

## Discussion

As the use of lasers for deterring birds becomes more widespread (Bishop et al., 2003; Vantassel and Groepper, 2015; Atzeni et al., 2016; Brown and Brown, 2021), it is critical to understand their effects on avian behaviours that can indirectly affect their survival. While previous studies have documented bird avoidance and movement in response to laser exposure (Glahn et al., 2000; Werner and Clark, 2006; Cassidy, 2015; Atzeni et al., 2016; Gorenzel et al., 2016; Manz et al., 2024), our study is the first to investigate the direct effects of laser exposure on bird visual exploratory and foraging behaviour through a manipulative approach with controlled dosage. After being exposed to a high power laser used as an avian deterrent (Seabird Saver), house sparrows approached the food patch quicker, reduced their use of binocular vision, developed a bias for using the left eye when visually exploring the food patch (particularly with higher laser energy levels), increased pecking rate and changed scanning rates depending on how conspicuous seeds were. Many of these changes began occurring at relatively low laser energy levels (1 mJ), and the changes became more pronounced above 3 mJ. These behavioural modifications after laser exposure are consistent with the idea that lasers could damage the eye, resulting in changes in visual function while foraging.

In studies that exposed mammalian eyes to lasers, behavioural tests show a loss of visual function due to eye injury, which in itself may or may not have been detected (Zwick, 1989; Zwick et al., 1994; Robbins and Zwick, 1996). How long an injury takes to develop after laser exposure depends on the damage mechanism: thermal or photochemical (Barkana and Belkin, 2000; Glickman, 2002). Thermal damage occurs when energy from a particular wavelength is absorbed by a chromophore in a cell and temperature in the cell increases faster than it can be dissipated (Thomsen, 1991; Barkana and Belkin, 2000). Temperatures rising in the cell causes protein denaturation and coagulation and leads to loss of cell structure or cell death (Birngruber et al., 1985; Thomsen, 1991). Photochemical damage is caused by long exposures of shorter wavelengths at low energy levels that do not increase cell temperature. Instead, these exposures induce chemical reactions that break down nucleic acids and lead to cell death over time (Barkana and Belkin, 2000; Glickman, 2002; Wu et al., 2006). Due to the complex nature of tissue and energy interaction, there is no clear boundary between the energies at which thermal and photochemical damage mechanisms operate, and instead both damage mechanisms may be operating (Zwick, 1984; Robbins, 1992; Glickman, 2002; Denton et al., 2007; Pocock et al., 2014). Subsequent morphological and structural injury due to both thermal and photochemical laser damage develops over hours to days (Moon et al., 1978; Matylevitch et al., 1998; Glickman et al., 2007), and functional changes in the eye could manifest as changes in visual behaviour over longer periods of time (Zwick, 1989; Zwick et al., 1997; DiCarlo et al., 2006). For instance, Robbins (1997) noted an increase in erratic behaviour and variability in visual acuity for days and even weeks after laser exposure in rhesus monkeys.

Acute damage to the retina, such as retinal oedema or swelling of the retinal tissue (Powell et al., 1971; Tso, 1973; Robbins, 1997; Barkana and Belkin, 2000; Paulus et al., 2008) can cause visual functional deficits (Randolph et al., 1983; Schmeisser, 1990), including visual acuity impairment (Zwick, 1984; Robbins, 1997). A systematic review of case studies in which 111 patients were evaluated for laser-eye injury from continuous wave laser pointers revealed that 55% had visual acuity deficits of 50% to 95% at initial presentation (Birtel et al., 2017). Additionally, studies on humans and non-human primates support the idea that laser exposure can change chromatic sensitivity and chromatic acuity (Robbins et al., 1980; Zwick et al., 2003), even if visual acuity remains at normal levels (Glickman et al., 1996). These changes can be the result of altered cell composition in the retina. Laser exposure can cause damage to or even death of photoreceptors and leave lesions or areas unpopulated by cell somata in the retina (Powell et al., 1971; Busch et al., 1999; Zwick et al., 2008). Interestingly, some studies found that photoreceptors appeared in spots that previously had consisted only of dead photoreceptors (Busch et al., 1999; Paulus et al., 2008; Zwick et al., 2008; Sher et al., 2013). There is no evidence of photoreceptors regenerating in mammals (Sher et al., 2013) or adult birds (Goldman, 2014); therefore, photoreceptors could be actively or passively migrating to these empty spots from other parts of the retina (Tso, 1973; Zwick et al., 1999, 2003, 2008), which would shift the way birds would use their centres of acute vision (i.e. foveae), including colour perception, and their binocular vision. Actually, Harris (2021) found different types of eye injuries (corneal oedema, cataracts, retinal atrophy, scleara degeneration) in the house sparrows exposed to the laser used in the present study.

We found changes in house sparrow foraging and visual behaviour that are contingent on the time since laser exposure. Birds arrived quicker to the food patch within Week 1 after laser exposure. One explanation for this is that laser exposure could have affected house sparrow visual acuity, which is 4.88 cycles/degree (Dolan and Fernández-Juricic, 2010), meaning individuals can see a 2-mm millet seed from over 1 m under ideal viewing conditions. The food patch was placed approximately 0.60 m away from the perch, well within their range of visual acuity. Following previous estimates on visual acuity reduction in humans exposed to lasers (50–95%; Birtel et al., 2017), we can estimate the potential consequences for house sparrows. A 50% reduction in visual acuity would result in a house sparrow only being able to resolve a 2-mm millet seed from 0.56 m away, whereas a 95% reduction in acuity would reduce that resolving distance to 0.056 m. A reduction in visual acuity may have decreased latency if birds had to get closer to the food patch to resolve visually the presence of seeds. Additionally, latency within Week 1 after exposure was also affected by the interaction between seed visual contrast and laser energy. As birds were exposed to energies higher than 1 mJ, it is possible that the physical damage to their retinas increased (Barkana and Belkin, 2000) making deficits in vision (i.e. trouble discriminating seeds) more pronounced (Weiskrantz and Cowey, 1967; Zwick et al., 1982; Robbins, 1997; Ben-shlomo et al., 2006; DiCarlo et al., 2006), which may have led to the decrease in latency in arriving to the food patch.

Alternatively, the changes in latency observed within Week 1 after laser exposure could be due to anaesthesia, which is known to create oxidative stress (Kotzampassi et al., 2009) and increase stress hormones (Zahl et al., 2010). Elevated corticosterone could have impacts on activity patterns, such as increased exploration and perch hopping (Haller et al., 1997; Breuner et al., 1998; Wingfield and Kitaysky, 2002). Supporting the idea of a stressful event for our experimental subjects (regardless of whether it was due to laser injury or anaesthesia) is the substantial decrease in body mass the days after exposure, followed by an increase in body mass towards pre-exposure levels over the following days.

Within Week 2 after laser exposure, the time it took birds to approach the food patch returned to pre-laser exposure levels. This behavioural change over time could be due to improvements in visual performance if the eyes began healing. In a study on the recovery of laser eye injury in rats, injuries were at their worst 24 to 48 hours after exposure and the healing process began 72 hours after exposure (Belokopytov et al., 2010). Another study reports that intense lesions in rabbits were reduced to 54% of their original size one week after laser exposure (Paulus et al., 2008). Improvements in visual acuity following laser exposure injury have also been documented (Zwick, 1984). However, we argue that the rapid improvement in foraging performance may be due to laser-related inflammatory changes (most notable causing corneal oedema, but also retinal oedema, that can cause temporary disturbances in visual acuity) rather than retinal cell regeneration or migration.

We found that after laser exposure, birds scanned significantly more when they could easily distinguish seeds from the visual background than when seeds were more challenging to resolve. If laser exposure decreases contrast sensitivity of the eye, our low contrast foraging trials may have become more difficult, potentially leading to reduced scanning behaviour to allocate more time to other foraging behaviours to help detect seeds. For example, Lawrence (1985) showed that when blackbirds fed on cryptic prey, twice as much time elapsed between consecutive head-up scans.

We also found that house sparrows changed visual exploratory behaviours associated with foraging tasks after laser exposure: individuals used more the foveal vision of their left eye over their right eye despite both eyes having been exposed to the lasers and no difference in cone densities between the eyes (Ensminger and Fernández-Juricic, 2014). Preference for one eye over the other is well documented in birds depending on the task (Franklin and Lima, 2001; Rogers, 2008; Templeton and Christensen-Dykema, 2008; Martinho III et al., 2014). Actually, house sparrows have shown lateralization of copulatory behaviour where males preferentially make cloacal contact with females on the left (Nyland et al., 2003). Lateralization in vision has been well documented in the context of foraging as well (Mench and Andrew, 1986; Valenti et al., 2003; Beauchamp, 2013), possibly because of the specialization of brain hemispheres for certain visual tasks (Güntürkün and Kesch, 1987; Parsons and Rogers, 1993). For example, birds may use either the right or left eye preferentially to aid in differentiating a stimulus from its surroundings (Andrew, 1988; Rashid and Andrew, 1989) or colour discrimination (Vallortigara, 1989; Vallortigara et al., 1996; Skiba et al., 2000). Following this logic, reduced quality of visual input in both eyes due to laser injury could have increased the difficulty of the foraging task leading house sparrows to use the brain hemisphere that is specialized for interpreting key foraging cues as a compensatory strategy.

Increased differences in the use of the left and right foveal vision may influence the use of binocular vision, which is thought to be used for close range, visually guided foraging to enhance prey detection and food handling (Martin, 2014). Binocular summation provides some advantages through enhanced acuity, contrast sensitivity, flicker detection, form recognition and visuomotor coordination (Blake et al., 1981; Kambanarou, 2005). For instance, starlings using binocular vision are better able to find inconspicuous prey (Templeton and Christensen-Dykema, 2008), and pigeons perform better in visual-discrimination tasks when allowed to use binocular vision (Watanabe et al., 1984). However, binocular summation plays a role when there is symmetry between the eyes (Marmor and Gawande, 1988; Pardhan and Gilchrist, 1992; Jiménez Cuesta et al., 2003; Castro et al., 2009; Pineles et al., 2013; Arba Mosquera and Verma, 2016). Furthermore, asymmetrical eye performance can even cause binocular inhibition, where binocular performance is worse than monocular vision (Pardhan and Gilchristt, 1990; Pardhan, 1993). A lack of binocular summation or binocular inhibition due to asymmetrical function of the right and left eye could explain why house sparrows decreased the use of binocular vision after laser exposure.

We also found that house sparrows actually increased their pecking rates and seed consumption rates (measured over 30 seconds) within Week 1 and within Week 2 after laser exposure. At first, this finding may be counterintuitive due to the aforementioned changes in foraging visual exploratory behaviour. One possibility is that these changes are related to the exposure to anaesthesia and the lower body mass, which would prompt a higher metabolic demand. Alternatively, if the laser injuries caused blurred vision and/or dark spots in the visual field, house sparrows may have compensated by increasing their foraging efforts to eventually peck on seeds that would have been more difficult to pinpoint visually. The increase in instantaneous foraging effort, along with an increase in seed consumption over 15 minutes within Week 2 after laser exposure, concurs with our body mass results, which show that over the days after laser exposure, individuals recovered their original body weight. A remaining open question is if the recovery in body mass may have fuelled healing of the eyes.

The rates at which birds pecked and consumed seeds were also affected by the interaction among laser exposure, seed visual contrast and laser energy. Within Week 1 after exposure, birds exposed to higher energy levels pecked and consumed seeds at higher rates in the high seed visual contrast. Reduced time searching for food and reduced visual performance with increasing laser energy could have led to birds increasing pecking rate to make up for the uncertainty that a given peck would be successful. Within Week 2 after laser exposure, birds pecked and consumed seeds at lower rates in the high seed visual contrast when exposed to higher laser energy and pecked and consumed seeds at higher rates in the low seed visual contrast when exposed to higher laser energy. This pattern could be explained by circumstances similar to those argued to affect latency. Decreased visual performance with increased laser energy exposure could have caused the low seed visual contrast task to become considerably more difficult than the high seed visual contrast. Increased difficulty discerning seeds from the background in the low seed visual contrast task may have increased pecking rates to counteract a difficult foraging task having been exposed to higher laser energies.

Although our findings can only be applied to house sparrows, there are some important conservation implications to be drawn for the health and survival of wild birds that are exposed to laser deterrents. Laser eye exposure to energies above 1 mJ could affect how quickly birds arrive to a foraging patch and how they forage and scan for predators, as a result of eye injuries (Harris, 2021), potentially compromising daily food intakes. This type of behavioural changes could increase energy expenditure and vulnerability to predators. This assumes that birds do not change their behaviour (blinking, moving the head away from the laser, leaving a patch) during laser exposure. This assumption has been corroborated in some bird species, but not in others (Blackwell et al., 2002). We note that our exposure times were very short (0.1 to 1 second) and that birds that do not divert their eyes away from a laser could potentially be exposed to longer times. Therefore, we recommend that conservation practitioners using handheld laser deterrents make every effort possible to avoid exposing the head of the bird and if accidentally done, change the direction of the exposure as soon as possible.

We have yet to assess the long-term consequences of laser exposure in birds. There is evidence in mammals that repeated exposure can have an additive effect on visual function and lead to permanent, long-term, as opposed to temporary, short-term, visual deficits (Griess and Blankenstein, 1981; Robbins and Zwick, 1996). Additionally, lasers used for bird deterrence come in many different wavelengths and powers, which might have different effects of avian physiology and behaviour (Lustick, 1973; Alaasam et al., 2018). Although we focused our attention on a high-power laser deterrent and a small songbird, similar deterrents can be used to disperse much larger birds that have different visual systems and foraging strategies (Glahn et al., 2000; Sherman and Barras, 2004; Werner and Clark, 2006; Gorenzel et al., 2016). Future research should include other laser units available in the market and, if ethically and logistically possible, species of management concern.

## Supplementary Material

Web_Material_coag004

## Data Availability

To make all statistical analyses and figures reproducible, we made the R code and data files available at the Open Science Framework (https://osf.io/3un8b/).
