# Supplementary material for: Effects of a high-power laser eye exposure on avian foraging behaviour: implications for the safety of laser bird deterrents
Source: Conserv Physiol. 2026 Feb 5;14(1):coag004. doi: 10.1093/conphys/coag004 (PMC12880189; doi:10.1093/conphys/coag004)
Supplement: Web_Material_coag004 [file web_material_coag004.docx]

**Appendices**

**Appendix 1**. Food patches and visual backgrounds

Food patches were constructed from standard Petri dishes (110 mm x 30 mm) (Fig. A1.1). We filled the Petri dishes with 32 g of TOHO seed beads (https://www.tohobeads.net/) as the substrate (bead size 11/0, hole size 0.7 mm).

We estimated chromatic contrast of the millet seeds against the different bead colors by using the receptor noise limited model (Vorobyev and Osorio 1998). From a visual physiology perspective, our model incorporated species-specific information on the house sparrow’s cone sensitivity, ocular media transmittance, and relative density of cones (Ensminger and Fernández-Juricic, 2014). We measured the reflectance of the millet seeds as well as the different types of beads using a JAZ portable spectrometer (Ocean Optics, Inc, Dunedine, FL, USA) sampling from 300 to 700 nm. We measured the ambient light properties inside the enclosures used in the experiment using the same spectrometer. Our chromatic contrast calculations followed Moore et al. (2012).

In the training phase, house sparrows learned how to forage for 15 millet seeds in the food patches with millet seeds that had chromatic contrast of 39 Just Noticeable Differences (JND) against the Matt Opaque Black TOHO seed beads (Fig. A1.2). In the experimental trials (before exposure to laser, within week 1 and within week 2 after laser exposure), each bird participated in a high seed visual contrast trial and a low seed visual contrast trial. The *high visual contrast* treatment used the “silver” substrate (TOHO gold lustered grey seed bead, size 11/0, hole size 0.7 mm), which was 34 JNDs contrast between the millet seed and the substrate (Fig. A1.2). The *low visual contrast* treatment was the “gold” substrate (TOHO frosted gold-lined crystal seed bead, size 11/0, hole size 0.7 mm), which was 6 JNDs contrast between the millet seed and the substrate (Fig. A1.2). In both visual contrast treatments, the seeds were above the accepted threshold of discrimination (1-4 JND) for the receptor noise limited model (Endler and Mielke 2005), which suggested that birds were not compromised visually in terms of detecting the seeds from the visual background. However, the high and low chromatic contrast treatments were expected to make it easier and more difficult, respectively, for the birds to visually resolve details of seeds relative to the substrate.

**Fig. A1.1:** Foraging patch made out of a Petri dish used in this study. The red arrows show the approximate projection of the centers of acute vision (foveal vision), and the blue lines display the approximate projection of the binocular field (binocular vision).


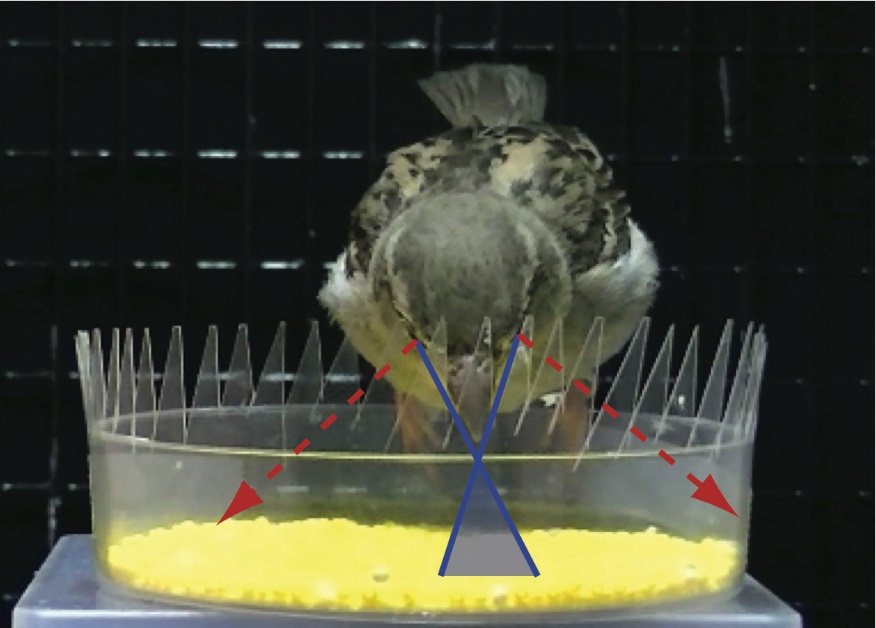


**Fig. A1.2:** Photographs (and chromatic contrast estimates in units of Just Noticeable Differences) of the millet seeds and the different backgrounds used in the training phase as well as the foraging trials.


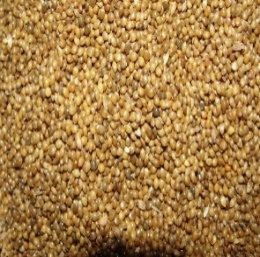


**15 millet seeds**


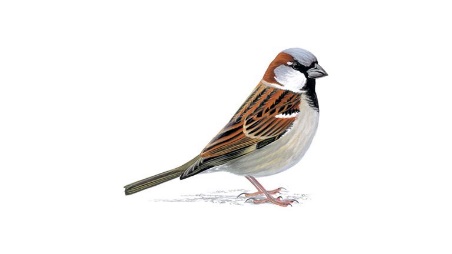

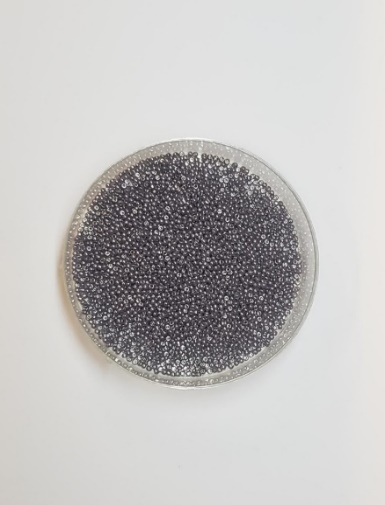


HIGH


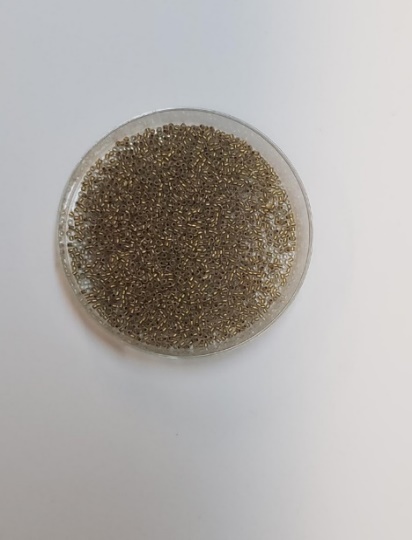

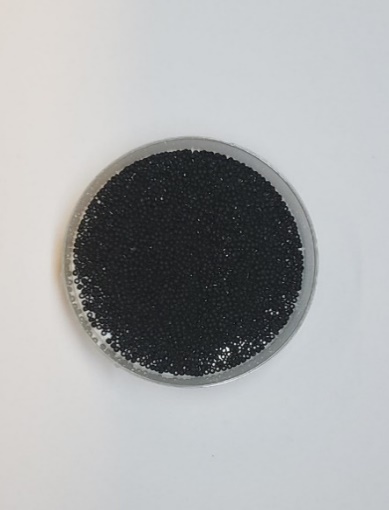


**Foraging trials**

**Training**

39 JND JNDJNDJND

6 JND

34 JND JND

**Seed visual contrast**

LOW

References cited:

Endler, J. A., and P. W. Mielke (2005). Comparing entire colour patterns as birds see them. Biological Journal of the Linnean Society 86:405–431.

Ensminger, A. L., and E. Fernández-Juricic (2014). Individual variation in cone photoreceptor density in house sparrows: Implications for between-individual differences in visual resolution and chromatic contrast. PLoS ONE, 9(11).

Moore, B.A., P. Baumhardt, M. Doppler, J. Randolet, B.F. Blackwell, T.L. DeVault, E.R. Loew & E. Fernandez-Juricic. 2012. Oblique color vision in an open-habitat bird: spectral sensitivity, photoreceptor distribution, and behavioral implications. Journal of Experimental Biology 215: 3442-3452.

Vorobyev, M., and D. Osorio (1998). Receptor noise as a determinant of colour thresholds. Proceedings of the Royal Society B: Biological Sciences 265:351–358.

**Appendix 2**. Phases in the experiment: details

*Training*

We trained birds how to forage for millet seed from a background substrate with beads to prepare them for the behavioral trails. Six randomly chosen birds at a time were housed in one cage for two consecutive days before the pre-exposure trials. When the lights went off at 21:00, we removed the six food patches containing food mix from the cage and weighed them. Lights were turned back on at 7:00. We returned the six food patches to the cage at 11 AM with 32 g highly contrasting black TOHO seed beads (size 11/0, hole size 0.7 mm) and between 10 and 30 millet seeds to acclimate birds to finding food on the substrate. The chromatic contrast of the millet seeds against the chosen background for the training trials was 39 Just Noticeable Differences (JNDs) from the house sparrow visual perspective (details of the visual contrast calculations in Appendix 1). We then left the room for 15 min to allow them to forage. When we returned to the room, we evaluated the success of the birds by noting if the millet seeds were eaten or remained untouched. If 50% or more of millet seeds were untouched, we considered this a failure and immediately gave birds a second chance. After the second try, we weighed each bird and replaced the beads with 80 g of food mix. We repeated this process the following day. After the second day of training for all bird groups, all food patches had at least 50% or more millet seeds eaten, and birds were considered ready to proceed to the before laser exposure trials.

*Before laser exposure trials*

To assess baseline foraging behavior, birds completed two pre-laser-exposure trials. After training, we transferred birds to individual cages in an experimental room (6 birds at a time as we were space-constrained). This room contained six 0.61 x 0.61 x 0.76 m mesh-wired enclosures designed specifically for our experimental procedure (details of the experimental arena are in Appendix 2). One side of the enclosure consisted of a 3.175 mm thick piece of acrylic, UV transparent Plexiglas that allowed us to video tape the bird using a high-definition camera (JVC GZ-E10BUS) secured 8 cm from the bottom of the cage on the other side of the Plexiglas. Food patches were placed in the cage so that birds could land on the smooth portion of the dish and face the Plexiglas wall and camera. On top of each cage was a full spectrum light (BluemaxTM Prolumne T8 fluorescent tubes, Model #109212, Full Spectrum Solutions, Inc., Jackson, MI), under a 12 hr light / 12 hr dark cycle from 0830 to 2030. The mesh-wire side of the enclosure opposite the camera was covered by a black curtain to create a contrasting backdrop. The remainder of the enclosure was covered with white curtain to limit visual and audible distractions for the birds.

Birds were food deprived overnight by removing the food patches from the cages at 20:30 and weighing the food. Lights turned back on at 8:30 and we began experimental trials at 9:00. Birds were tasked with finding 15 millet seeds on a substrate of TOHO seed beads of either “high” or “low” chromatic contrast from the visual perspective of house sparrows. Each bird was randomly assigned to either the high or low (“silver” or “gold”) seed visual contrast and completed one trial per day. We left the room and video-recorded the trial for 15 minutes, then retrieved the food patches and recorded the number of seeds eaten by the bird. We continued this process for each bird. If birds did not participate (no seeds eaten), we allowed them to make another attempt. If birds did not participate in the trial by 13:00 hrs, they were removed from the experiment. After all willing birds completed their trial, we weighed each bird and returned the food patches to the cages with 80 gr of food mix. We repeated these procedures for a second night/day but presented individuals with the substrate (either high seed visual contrast or low seed visual contrast) they were not given in the first trial. By holding the second trial the next day as opposed to later on, we were able to keep the hunger level and motivational conditions of both trials consistent.

*Laser exposure*

After the pre-laser exposure trials, we weighed and transferred the birds individually to another room in the Purdue animal care facility. We administered 20 µL of refrigerated rocuronium bromide to each eye to dilate the bird’s pupils. This dosage was recommended by Dr. Townsend of Purdue University Veterinary Hospital. It took approximately 30 min for pupils to fully dilate. Birds were then anesthetized to eliminate small ocular movements that could alter the amount of laser light entering the eye and to reduce stress. We initially based the dosage of our anesthesia solution off Velez et al. (2015), but adjusted it to 4 mg/kg midazolam, 8 mg/kg ketamine and 2 mg/kg xylazine based on conversations with veterinary staff. Using an aseptic technique and training from Purdue Animal Care and Use Committee, we injected the anesthesia solution into the bird’s breast muscle. We gently transferred anesthetized birds to a different room inside a bag on a microwaved heating pad and several layers of towels.

The laser exposure room was set up with the training and approval of Purdue Radiological and Environmental Management and all persons in the room followed proper safety protocol by wearing safety eyewear appropriate for the laser unit used. Multiple laser models are currently available to deter birds outdoors. All emit wavelengths that we perceive as either red or green light and are continuous wave lasers, which means they deliver a constant energy. Class II lasers emit powers below 1mW and are not considered a hazard when viewed for 0.25 seconds (the human aversion response) or less (ICNIRP, 2000; Ziegelberger, 2013*b*; American National Standards Institute, 2014). Class IIIA lasers include any devices that emit between 1 and 5 mW power. Class IIIB lasers are those that range from 5-500mW power and can be hazardous if viewed directly for any period of time. However, there are laser units available that exceed 500 mW, such as the laser prototype used in our study. Lasers operating over 500 mW are labelled as class IV and are considered by OSHA to be hazardous under any viewing condition, including diffuse viewing and viewing of reflections (Occupational Safety and Health Administration Office of Science and Technology Assessment, 1995; ICNIRP, 2000; American National Standards Institute, 2014). The laser we used, a prototype of the Seabird Saver (https://www.bmis-bycatch.org/mitigation-techniques/seabird-saver), had adjustable wattage (from 0 – 1,000 mW), had a beam diameter of 4 cm at the aperture, beam divergence of 0.5 mrad, gaussian beam shape, and 532 nm wavelength. The reason we chose this laser unit is because Federal authorities were interested in assessing whether it could cause an effect on bird behavior given its increasing use in the fishing industry. The laser unit was taped securely on a table and fitted with a Thor labs 1-in optical beam shutter and shutter controller attached so the new laser aperture was 2.54 cm. Exactly 1 m from the laser aperture, we placed a power sensor (Ophir 30A-BB-18 power sensor). We visually aligned the center of the power sensor with the laser beam by adjusting the height of the meter and moving the meter either left or right. When the reading from the power meter (Ophir Vega laser power meter) was the power desired for exposure, we marked the location of center of the meter then moved the power meter approximately 6 cm directly backwards (details of the laser exposure setup in Appendix 3)

We strapped each bird into a foam cradle using Velcro straps and secured their feet. We placed the restrained bird on the marked location in front of the power meter exactly 1 m from the laser aperture such that one eye of the bird was centered with both the power sensor and laser beam. The eye facing the beam was temporarily secured open and exposed the bird to the appropriate power level and duration three times. We chose three exposures in order to replicate the likely conditions birds would experience in the field (E.F. Melvin personal observation). We waited three seconds between exposures (recommended by Bruce Stuck, Director of the Ocular Trauma Research Division at the U.S. Army Institute of Surgical Research in San Antonio, Texas until 2013) in order to prevent possible additive effects (Thomsen, 1991; Lund and Sliney, 2014) and repeated the same exposure procedure on the opposite eye. After both eyes were exposed, we removed the bird from the cradle and placed it back in the bag over a warmed pad to maintain its body temperature. We monitored birds until they were awake (between 30 min and 3 h) and returned them to their individual cages with 80 gr food and water ad libitum.

Each of the 40 birds used in the study was exposed to a single energy level. We consequently used 40 different energy levels to explore how a relatively broad range could influence foraging behavior. Because no previous studies have determined laser injury thresholds in birds, we based our range of energies on the accepted human laser safety guidelines (American National Standards Institute, 2014), which in turn stem from controlled experiments mostly in non-human primates (Farrer et al., 1970; Lund et al., 2007). In these experiments, the eye is exposed to incremental dosages and assessed for signs of damage (Zwick et al., 1994; Lund et al., 2007). Based on the American National Standard for Safe Use of Lasers and the International Commission on Non-Ionizing Radiation Protection (American National Standards Institute, 2014), the threshold for laser damage is the dose at which an individual has a 50% probability of having damage. Our goal was to empirically assess the actual injury threshold (observed ED50) by estimating the range of predicted ED50s for birds and using that range as a guideline. We then used the predicted ED50 values as median doses and expose birds to values 3 times below the lowest predicted ED50 and 3 times above the highest predicted ED50. The rationale behind this strategy was to have a wide range of energy values to assess the behavioral consequences of laser exposure. First, we calculated the maximum permissible exposure (MPE), which is one tenth of the ED50 (American National Standards Institute, 2014). Like the ED50, the MPE can be expressed as the radiant energy per unit area in $\frac{mJ}{cm^{2}}$ , also called corneal irradiance (American National Standards Institute, 2014). We used the following equation to calculate the MPE of continuous wave lasers (like our unit) that are 400-700 nm for laser exposure times between 5 µs and 10 s:

$MPE=1.8*t^{0.75}$;

where t is laser exposure time in s (American National Standards Institute, 2014). The 7 laser exposure times (0.1, 0.25, 0.4, 0.55, 0.7, 0.85, 1.0 s) that we chose were based on times found in the literature that we could reach with our equipment (Leibu et al., 1999; Ham et al., 1970). The equations we used are based on a human pupil diameter of 7 mm, so we corrected them for a 2.22 mm house sparrow pupil. We corrected the MPE values by multiplying them by the ratio (approximately 5.52) of human pupil area (38.48 mm2) to house sparrow pupil area (6.97 mm2, measured on a dilated eye). This gave us a range of MPE values from 1.77 mJ/cm2 to 9.94 mJ/cm2 that we then multiplied by 10 to get the predicted ED50’s. We divided the ED50s by 3 because we planned to expose each eye 3 times. The final predicted ED50 values, which represented our predicted threshold of laser eye injury for house sparrows when exposed to a laser 3 times for 0.1 to 1s, ranged from 5.89 to 33.12 mJ/cm2. We then estimated the corneal irradiances that were approximately 3 times below the lowest predicted ED50 and 3 times above the highest predicted ED50 to obtain our final irradiance values. Our range of corneal irradiances were 1.96 to 99.36 mJ/cm2. The next step was to apply the final irradiances using the laser, which required the manipulation of two factors: laser power and laser exposure time. We used the following mathematical relationship that we modified from the ANSI guidelines (American National Standards Institute 2014):

$\left( \frac{\mathrm{Power}mW}{Beam Area at Cornea{cm}^{2}} \right)*Time s=Corneal Irradiance\frac{mJ}{{cm}^{2}}$;

where the beam area at cornea was 5.07 cm2, based on the 2.54 cm diameter of the shutter we fitted to the laser. We chose 7 laser powers (60, 90, 130, 165, 200, 235, 270 mW) to substitute into this equation that were low compared to our possible range of powers (0-1000 mW) and would give us final irradiances that were within the range we wanted. We assumed beam size did not change from aperture to the cornea because the beam divergence was low at 0.5 mrad. Using the 7 powers and times mentioned earlier, we calculated 49 different irradiances ranging from 1.15-53.2 mJ/ cm2. We then converted them to total intraocular energies (TIEs) by multiplying the values by the area of a 2.22 mm house sparrow pupil, which gave values in mJ. The final energies for the house sparrows ranged from 0.08-3.71 mJ (0.08, 0.13, 0.18, 0.21, 0.23, 0.27, 0.32, 0.33, 0.37, 0.45, 0.52, 0.57, 0.58, 0.69, 0.70, 0.71, 0.72, 0.81, 0.82, 0.91, 0.93, 1.10, 1.11, 1.25, 1.31, 1.48, 1.51, 1.59, 1.78, 1.79, 1.92, 1.93, 2.04, 2.26, 2.27, 2.34, 2.60, 2.75, 3.16, 3.23, 3.71 mJ).

*After laser exposure trials*

The birds participated in the after-exposure trial following the same procedure as the before laser-exposure trials approximately 24 h after laser exposure, and again the day after that, approximately 48 h after exposure (two trials to expose them to the low and high food visual contrast conditions randomly). We called these two trials “within week 1” trials. Seven and eight days after the laser exposure the birds participated in another set of two after-exposure trials (with low and high food visual contrast conditions presented randomly). We called these two trials “within week 2” trials. Due to an error in planning for within week 2 trials, 3 out of the 40 birds had their second after-exposure trail on the tenth day after-exposure. After the within week 2 trials, birds were weighed and euthanized with CO_2_.

**Fig. A2.1:** (A) side view (B) top view of experimental cage setup


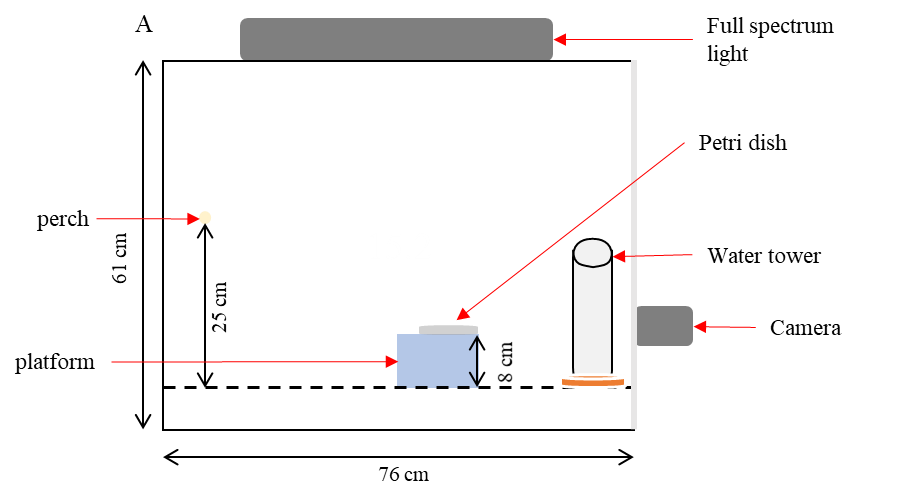

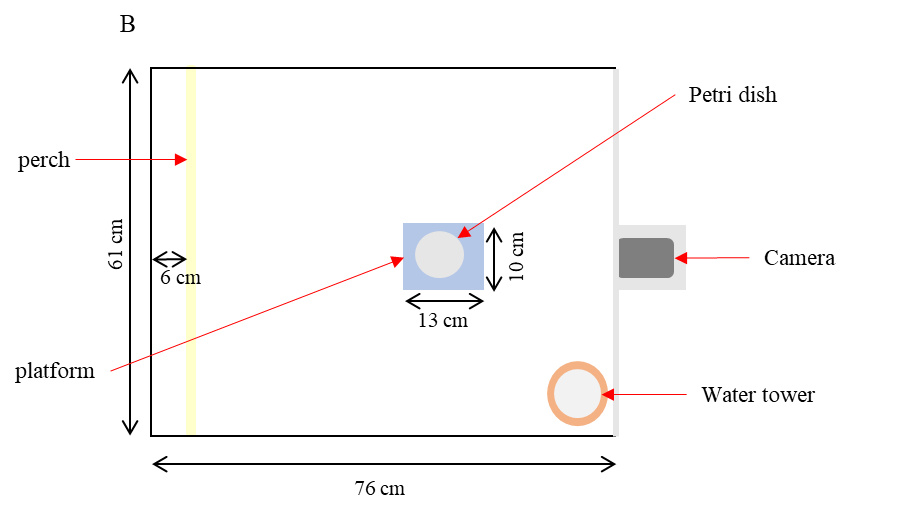


**Appendix 3.** Laser exposure setup.

Each bird was placed in a foam cradle exactly 1 m from the aperture of the laser, which was secured on a table and fitted with a Thor labs beam shutter 2.54 cm diameter and 15.24 cm behind the bird was an Ophir power sensor which was aligned to the center of the laser beam.

**Fig. A3.1:** Schematic representation of the positioning of the bird and the laser when animals were exposed to the laser beam.


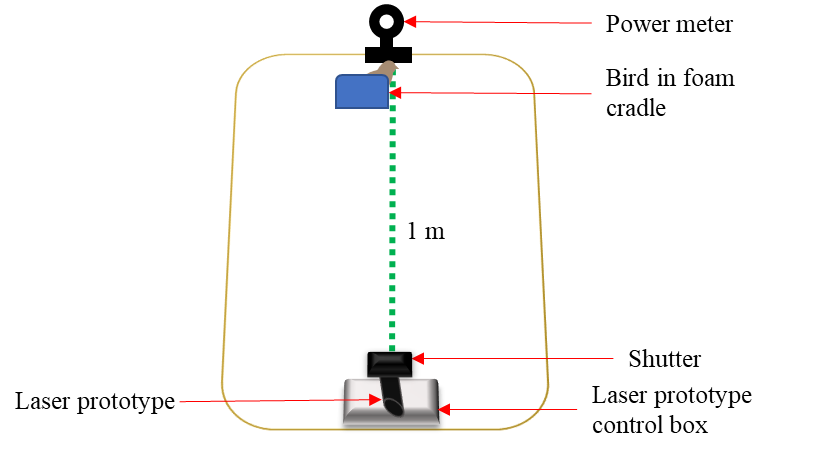


**Appendix 4.** List of behaviors coded for in BORIS using frame by frame function

| **Behavior Coded** | **Definition of behavior** |
| --- | --- |
| Start | Trial begins when the experimental cage door closes |
| End | Trial ends when the bird has been participating (at the food patch) for 30 seconds |
| Arrive | Both feet make contact with the dish or platform |
| Leave | Both feet are no longer in contact with the dish or platform |
| Peck | Bird makes head movement toward dish and beak makes contact with substrate or seed |
| Seed consumption | Bird successfully captures seed. Seed seen in beak accompanied by chewing or husk flying |
| Scan 1 | Beak is above the horizontal and head is not tilted or head is turned away from the dish so that the beak is not projecting into the food patch |
| Scan 2 | Beak is below horizontal but does not project into the food patch and head is not tilted |
| Binocular vision | Beak projects into the food patch and head is not tilted |
| Binocular-foveal vision | Beak projects into the food patch and head is tilted so that both eyes can still see into the food patch |
| Foveal vision | Beak does not project into dish and head is tilted so that only one eye can see the food patch |
| Cannot tell | Bird’s head and/or beak is blocked or cannot be determined |
